# Engineering an electroactive *Escherichia coli* for the microbial electrosynthesis of succinate from glucose and CO_2_

**DOI:** 10.1186/s12934-019-1067-3

**Published:** 2019-01-28

**Authors:** Zaiqiang Wu, Junsong Wang, Jun Liu, Yan Wang, Changhao Bi, Xueli Zhang

**Affiliations:** 1 Center for Molecular Metabolism, School of Environmental and Biological Engineering, Nanjing University of Science and Technology, Nanjing, 210094 China; 2 Key Laboratory of Systems Microbial Biotechnology, Tianjin Institute of Industrial Biotechnology, Chinese Academy of Sciences, 32 West 7th Ave, Tianjin Airport Economic Park, Tianjin, 300308 China

**Keywords:** Microbial electrosynthesis, Bioelectrochemical systems, Succinate, CO_2_ fixation

## Abstract

**Background:**

Electrochemical energy is a key factor of biosynthesis, and is necessary for the reduction or assimilation of substrates such as CO_2_. Previous microbial electrosynthesis (MES) research mainly utilized naturally electroactive microbes to generate non-specific products.

**Results:**

In this research, an electroactive succinate-producing cell factory was engineered in *E. coli* T110(pMtrABC, pFccA-CymA) by expressing *mtrABC, fccA* and *cymA* from *Shewanella oneidensis* MR-1, which can utilize electricity to reduce fumarate. The electroactive T110 strain was further improved by incorporating a carbon concentration mechanism (CCM). This strain was fermented in an MES system with neutral red as the electron carrier and supplemented with HCO_3_^+^, which produced a succinate yield of 1.10 mol/mol glucose—a 1.6-fold improvement over the parent strain T110.

**Conclusions:**

The strain T110(pMtrABC, pFccA-CymA, pBTCA) is to our best knowledge the first electroactive microbial cell factory engineered to directly utilize electricity for the production of a specific product. Due to the versatility of the *E. coli* platform, this pioneering research opens the possibility of engineering various other cell factories to utilize electricity for bioproduction.

**Electronic supplementary material:**

The online version of this article (10.1186/s12934-019-1067-3) contains supplementary material, which is available to authorized users.

## Background

Energy is a sine qua non of all biosynthetic processes, and is supplied in the form of chemical energy from the metabolism of substrates or from light through photosynthesis. Moreover, certain microbes were recently found to be able to utilize electric energy for the synthesis of chemical compounds [1–3]. The process in which microorganisms utilize electrons from a cathode to reduce carbon dioxide, glucose or other substrates is termed microbial electrosynthesis (MES) [4]. Due to the necessity for the reduction of CO_2_ emissions and storage of various forms of renewable electricity, research on microbial electrosynthesis techniques is becoming increasingly popular [1, 5–7].

Microbial electrosynthetic processes are performed in a bioelectrochemical system (BES) for biological reductive reactions, which comprises an anode, a cathode and a reference electrode [8, 9]. Electrons derived from the cathode are transformed into reducing equivalents to power biological processes such as fumarate reduction or CO_2_ fixation, while an oxidation process occurs at the anode at the same time [10–12]. When the electrons derived from the cathode are used by the fermentative microbial catalysts, the fermentation balance shifts toward the production of more reduced metabolic products [4, 10, 11, 13]. More importantly, it may be possible to reduce CO_2_ and fix it into organic compounds using the reduction power stemming directly from electric energy.

Current MES experiments are mostly based on naturally electroactive microbes, such as *S. oneidensis* MR-1 [14–16]. While these microbes have high electric activity, the palette of products that can be synthesized is limited to simple compounds such as acetate and formate [10, 17]. Furthermore, there are no mature synthetic biology tools available for these microbes, precluding their engineered into applicable cell factories. Thus, more versatile MES platforms based on model bacteria are needed to expand the capacities of the MES technique.

In this study, the electrically-inactive model microbe *Escherichia coli* was used to study the key factors involved in microbial electric activity, and was engineered into an electroactive bacterium to perform MES. The electron transfer pathway of *S. oneidensis* MR-1, which consists of a *c*-type outer membrane cytochrome (MtrC), a periplasmic *c*-type cytochrome (MtrA), a non-heme outer membrane β-barrel protein (MtrB), and an inner-membrane associated quinol oxidase (CymA) [14, 18], has been established as a model for biological electron transfer [17]. Therefore, we employed the electron transfer pathway from *S. oneidensis* in *E. coli* to make it electrically active and able to utilize electric energy in a biosynthetic pathway for CO_2_ fixation and production of reductive fermentation products.

## Materials and methods

### Strains, media, and growth conditions

The strains and plasmids used in this study are listed in Table 1. *E. coli* was cultured at 37 °C in Luria broth (10 g/L Difco tryptone, 5 g/L Difco yeast extract and 10 g/L NaCl). Apramycin Sulfate (50 mg/L; Ruitaibio, China), chloramphenicol (34 mg/L; Solarbio, China), ampicillin (100 mg/L; Solarbio, China), Streptomycin (50 mg/L; Solarbio, China) or β-d-1-thiogalactopyranoside (IPTG,1 mmol/L; Solarbio, China) were used where appropriate.

**Table 1:** *E. coli* strains and plasmids used in this research

Strains and plasmids	Characteristics	Sources
ATCC8739	Wild type	Lab collection
MG1655	Wild type	Lab collection
T110	ATCC8739, ^a^∆*ldhA*, ∆*pflB*, ∆*ptsI*, Ppck*-*galP*, Ppck*-*pck*	Tan et al. [46]
pACYC184	Cm, p15A origin of replication, *lacI*	Lab collection
pTrac99A-apr	Apr, pMB1 origin of replication, *lacI*	Lab collection
pTrac99A-spe	Str, pMB1 origin of replication	Lab collection
pTrac99A-M	Amp, pMB1 origin of replication, *lacI*	Lab collection
pBTCA	carbonic anhydride (CA) encoding gene (*ccaA*) and bicarbonate transporter (BT) encoding gene (*bicA*) in pTrac99A-M	Lab collection
pBAD-rfp	Kan, pBBR1 origin of replication, BBa J23100	Lab collection
pMtrABC	pACYC184 derived plasmid expressing the outer membrane protein complex encoding genes *mtrA*-*mtrB*-*mtrC* from *S. oneidensis*	This study
pFccA-CymA	pTrac99A-apr derived plasmid expressing cymA and *fccA* from *S. oneidensis*	This study
pFccA	pTrac99A-spe and pTrac99A-M derived plasmid expressing *fccA* from *S. oneidensis*	This study
pMtrA-RFP	pACYC184 derived plasmid expressing the fusion protein of MtrA-RFP	This study
pCymA-RFP	pTac99A-apr derived plasmid expressing fusion protein of CymA-RFP	This study
pFccA-RFP	pTrac99A-apr derived plasmid expressing fusion protein of FccA-RFP	This study

^a^△ represents the inactivated genes by gene deletion

* Represent the activate genes by gene regulation

### Construction of plasmids and strains

The *mtrA, mtrB,* and *mtrC* genes were amplified from *S. oneidensis* MR-1. The primers pACYC184-K-F and pACYC184-K-R were used for amplifying the plasmid backbone of pMtrABC, including the *lacI* gene and the *trc* promoter region. Artificial regulatory part RBS1 was embedded within the primers MtrB-F and MtrC1-F, respectively. The plasmid backbone and DNA fragments were ligated using Golden Gate DNA assembly [19] to obtain the plasmid pMtrABC. Similarly, the *fccA* and *cymA* genes were cloned from *S. oneidensis* MR-1 and ligated with a pTrac99A-apr backbone using the primers pTrac99A-K-F and pTrac99A-K-R to obtain pFccA-CymA, artificial regulatory part RBS2 was embedded within the primer CymA-F. To construct pFccA, the backbone of the plasmid pFccA was amplified using the primer pair pTrac99A-spe-F and pTrac99A-spe-R from pTrac99A-spe, the *lacI* gene was obtained from plasmid pTrac99A-M using primers pTrac99A-M-F and pTrac99A-M-R, the *FccA* gene was amplified from the genome of *S. oneidensis* MR-1, these three fragments were ligated using Golden Gate assembly. *E. coli* DH5α (CWBIO, China) was used for cloning and plasmid propagation, while T110 and 8739 served as the chassis strains. pMtrABC and pFccA-CymA have different resistance markers and origin of replication, and are compatible. Primers used in this study are listed in Additional file 1: Table S1.

### Microbial electrosynthesis reaction setup

A single colony of the relevant strain was used to inoculate 3 mL of fresh LB medium and cultured overnight at 37 °C under constant orbital shaking at 200 rpm. The resulting seed culture was used to inoculate fermentation medium at 2% (v/v), and anaerobically incubated at 30 °C and 200 rpm in 500-mL (183 mm × 108 mm × 36 mm) anaerobic flasks (ShuNiu, China). After 3 h of cultivation, the culture was induced with 0.1% (v/v) of a 1.0 mM isopropyl β-d-1-thiogalactopyranoside (IPTG) solution, sealed, and incubated for another 16 h under the same conditions. After measuring the OD_600_, the cells were harvested by centrifugation at 3000×*g* and 15 °C for 5 min. The obtained cell pellet was washed twice with NBS medium (New Brunswick Scientific, USA) and transferred into 99.99% pure platinum electrode clip of three-electrode bioelectrochemical reactors (Wenoote, China) for the microbial electrosynthesis reaction.

**Fig. 1:**
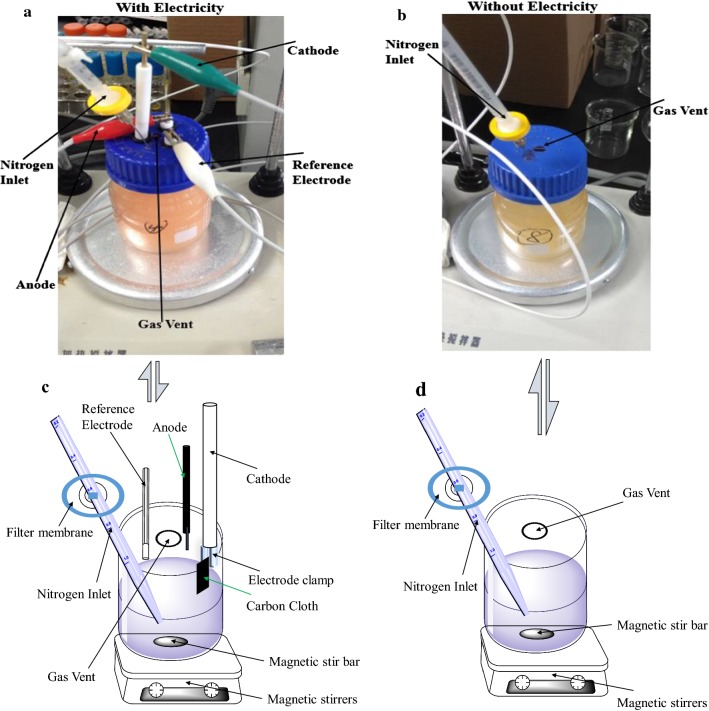
The *E. coli* MES reaction chamber constructed and used in this research. **a** The MES chamber supplied with electricity. The white electrode represents the reference electrode, the green electrode is the cathode, and the red electrode is the anode. A needle equipped with a sterile 0.22 μm filter (yellow) is the nitrogen inlet, and there is a gas vent between the three electrodes. **b** The MES chamber without electricity supply used for the control experiments. **c** The schematic diagram of the MES chamber supplied with electricity. **d** The schematic diagram of the MES chamber without electricity supply used for the control experiments.

The BES chamber was filled with 100 mL of modified NBS (New Brunswick Scientific) buffer solution composing (mmol/L): 25.72 KH_2_PO_4_, 28.71 K_2_HPO_4_, 26.50 (NH_4_)_2_HPO_4_, 1.00 MgSO_4_∙7H_2_O, 0.015 Thiamine HCl, 1.00 Betaine-KCl, with 27.78 or 55.56 glucose. The initial pH of the medium was 7.20, with 100 mM KHCO_3_ addition to provide CO_2_ as the carbon source for microbial electrosynthesis. The composition of MES reaction medium used for electroactivity experiment was same as the above mentioned modified NBS buffer, except that no KHCO_3_ or glucose was presented, but 10 mmol/L fumarate was added as electron receptor. The prepared samples were inoculated with an initial OD_600_ of 2.0–2.5. The working electrode’s (cathode) initial potential was controlled at − 650 mV_Ag/AgCl_ [4], sampling time was set at 7 days, and sensitivity (A/V) was set at 1.0 × 10^−3^. The O_2_ in the anaerobic medium was removed by constant sparging with pure N_2_ to ensure strict anaerobic conditions until the end of MES. The background current was monitored to ensure it is between − 5 and 0 μA, and the cyclic voltammograms were smooth, showing no significant peaks or waves. After 100 μL of a 10-mM neutral red solution (Sigma, USA) was added into the reaction medium, glucose was added to an initial concentration of 27.78 or 55.56 mM to initiate the MES process. The current was steady between − 10 and − 5 μA. The control group without neutral red showed only background current, demonstrating that there was no intrinsic electron transfer mediators in the medium. BES bioreactors without electrodes were used as negative controls. During the BES reactions, the current formed by the cathode that delivered electrons to the culture was monitored using a CHI1030C eight-channel potentiostat (Chenhua Instruments Co., Ltd., Shanghai, China). The BES platform for *E. coli* used in this research is shown in Fig. 1. Figure 1a and c represent the bioreactor with electricity, the green electrode is the working electrode (cathode), the red electrode is the anode, and the white electrode is the reference electrode. In the three-electrode reactor, the electrodes were linked with the potentiostat for electric control and measurements. The cathode was the working electrode, Ag/AgCl was the reference electrode, while the anode was the counter electrode. All potentials were reported with respect to the Ag/AgCl reference electrode. There was a gas vent for gas output between the positions of the three electrodes, as well as a gas inlet equipped with a needle and a 0.22 μm filter for delivering the high purity nitrogen. Figure 1b and d illustrates the BES bioreactor without electricity input. This negative-control reactor only had the gas vent and the inlet.

Control, measurement and analysis of the electric parameters were conducted using a CHI1030C eight-channel potentiostat (Chenhua Instruments Co., Ltd., Shanghai, China). A CHI115 platinum wire electrode and a CHI111 Ag/AgCl electrode (Chenhua Instruments Co., Ltd., Shanghai, China) were used as the counter electrode and reference electrode, respectively. A 2 × 2 cm carbon cloth pretreated with an 8% H_2_SO_4_ solution was nipped using the platinum electrode holders, and was used as the working electrode. The BES was operated in batch mode at room temperature (25 °C), in a 100-mL blue cap bottle (99 mm × 57 mm × 30 mm × 40 mm, ShuNiu, China). The three electrodes were inserted into the bottle via the cap. Pure nitrogen (N_2_) gas was sparged continuously into the reaction medium in the BES to maintain anaerobic conditions, while the BES chamber was stirred using a magnetic stirrer at 200–250 rpm.

### Membrane proteins extraction and samples preparation

To collect membrane-associated MtrA, MtrB, MtrC, CymA and FccA, cell membrane was extracted with the protocol: (1) Prepare the 500 mL-fermentation medium of the strains *E. coli* T110 and T110(pMtrABC, pFccA-CymA), then the cells are harvested by centrifugation at 3000×*g* for 10 min, (2) the cell pellet is redissolved using 15 mL PBS buffer (pH 7.0), followed by cell crushing for three times using the high-pressure homogenizer (JNBIO-3000 PLUS, China), (3) the cell suspension is centrifuged at 8000×*g* for about 60 min, (4) discard the pellet and collect the supernatant for the next step, (5) the membrane proteins are collected by centrifugation at 200,000×*g* using the ultracentrifuge (Optima L-100XP, Beckman, USA). The preparation of protein sample was as follows: (1) the collected membrane protein is dissolved using the 2.5 mL protein lysate (8 M urea, 1% DTT) and mixed well, (2) the suspension is centrifuged at 10,000×*g* for 15 min, 18 °C, (3) collect the supernatant into the 2 mL centrifugal tube and repeat this step once, and the samples are stored at − 80 °C for analysis or mass spectrometry.

### Analytical techniques and chemical analysis

Samples comprising 1 mL of the reaction mixture were obtained, and the supernatants were used for the analysis of the relevant metabolites including organic acids and residual glucose, using an Agilent 1260 series high performance liquid chromatography (HPLC) system (Agilent, USA), equipped with a refractive index detector and a Bio-Rad Aminex HPX-87H ion exclusion column (300 mm × 7.8 mm, Aminex, USA), which was kept at 35 °C and eluted with 5 mM H_2_SO_4_ at a flow rate of 0.5 mL/min; the injection volume was 20 μL [20]. The cathode was poised at − 650 mV in all the experiments. The curve of current versus time (i–t curve) was used to detect current changes during the entire process of BES reactions. The SDS-PAGE was run using the commercially purchased SurePage™ Gels (GenScript, Nanjing). The protein mass spectrometry was performed using the OrbiTrap Fusion LUMOS Tribrid Mass Spectrometer (LC–MS) (Thermo Fisher, USA) and the methods could be referred to references [21, 22].

### Statistical analysis

The significance of differences between mean values of control and test samples was compared by Student’s *t*-test, using the open-source software “R” (http://cran.r-project.org/). Differences with *p *< 0.05 were regarded as obvious, *p *< 0.01 as significant, and *p *< 0.001 as very significant.

## Results and discussion

### Design and setup of a neutral-red-mediated MEC system for *E. coli*

**Fig. 2:**
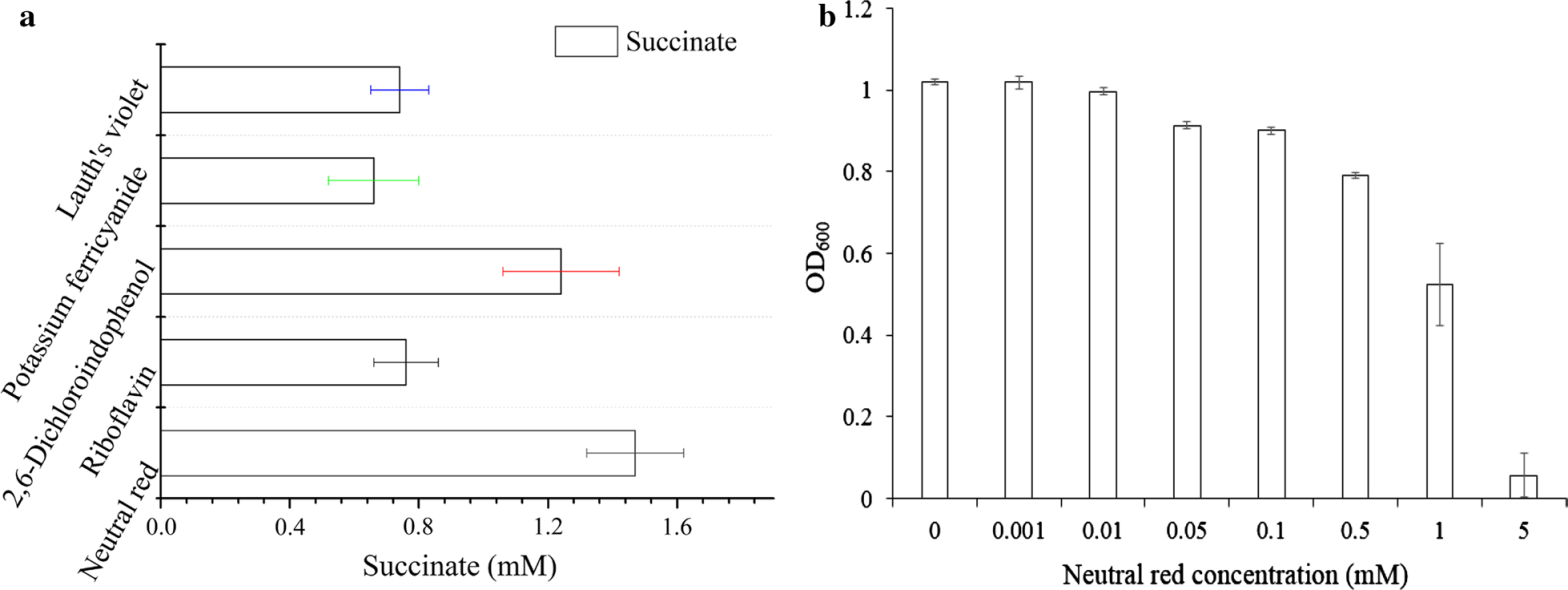
The determination of optimal electron carrier and concentration of neutral red. **a** Succinate production by *E. coli* T110 in MES with different electron carriers with fumarate as the substrate. **b** The optimal concentration determination of the electron carrier of neutral red by *E. coli* T110. The data represent the means of three parallel replicates and the error bars represent the standard deviation

In this study, we built an MES system according to previous reports (Fig. 1 and Additional file 1: Fig S1) [1, 10, 23]. In this system, the cathode was the working electrode, which gave out electrons to electron carriers, which in turn transferred the electrons to the microbes in the media. The other two electrodes were the Ag/AgCl as the reference electrode, and the anode as the counter electrode. To test the function of the MES system and screen for an appropriate electron carrier for subsequent experiments, neutral red, riboflavin, 2,6-dichloroindophenol, potassium ferricyanide, and Lauth’s violet were tested in a simple MES reaction in this system. The MES reaction used for testing was a simple one-step fumarate reduction reaction in the cells, in which a fumarate molecule accepts two electrons and is reduced to succinic acid. With 10 mM fumarate as a reaction substrate, the amount of the produced succinate was measured as an indicator of electron transfer efficiency. Although all carriers showed low efficiency, the results showed that a relatively high production of succinic acid could be achieved with neutral red as the electron carrier (Fig. 2a). Therefore, the designed MES was shown to be functional, and neutral red was adopted as the model electron carrier in this study.

Due to the reported toxicity of neutral red to living cells [24], the optimal concentration was determined to perform MES for *E. coli* T110. With the OD_600 nm_ value as the evaluation index of living cell number, various concentrations of neutral red (0, 0.001, 0.01, 0.05, 0.1, 0.5, 1.0 and 5.0 mM) were incubated with *E. coli* T110 with an initial OD of 0.5 for 12 h. Based on the results (Fig. 2b), when the neutral red concentration was in the range of 0.05, 0.1 and 0.5 mM, the cell growth was affected corresponding to the concentrations. In the range of 1 and 5 mM, the cell growth was significantly affected. And in the range of 0, 0.001 and 0.01 mM, the cells could keep up the growth. We have also done the same experiment with *E. coli* 8739, and the experimental result was as shown in Additional file 1: Fig. S3. *E. coli* 8739 exhibited a very similar pattern as the *E. coli* T110 with different concentrations of neutral red, except the final OD_600_ values were generally higher than that of *E. coli* T110 in the range of 0, 0.001 and 0.01 mM. Therefore, the optimal neutral red concentration for both strains was 0.01 mM.

Based on the previous experimental results of optimization of neutral red concentrations, the neutral red with a concentration higher than 0.01 mM was toxic to *E. coli* cells. We selected the neutral red concentrations below 0.01 mM including 0.005 and 0.01 mM, but also above 0.01 mM including 0.02, 0.03 and 0.04 mM, to investigate the effect of different neutral red concentrations on the current with strain *E. coli* T110 (pMtrABC, pFccA-CymA). The experimental results were shown as in Additional file 1: Fig. S4, that the current increased significantly when the neutral red concentration was raised from 0.005 to 0.01 mM. But in the range from 0.01 to 0.04 mM, the increasing extent was small. Considering both cell growth status and system current level, 0.01 mM neutral red was selected to perform the MES experiments in this work.

### Engineering of an electrosynthetically active *E. coli* with the key elements from *S. oneidensis*

**Fig. 3:**
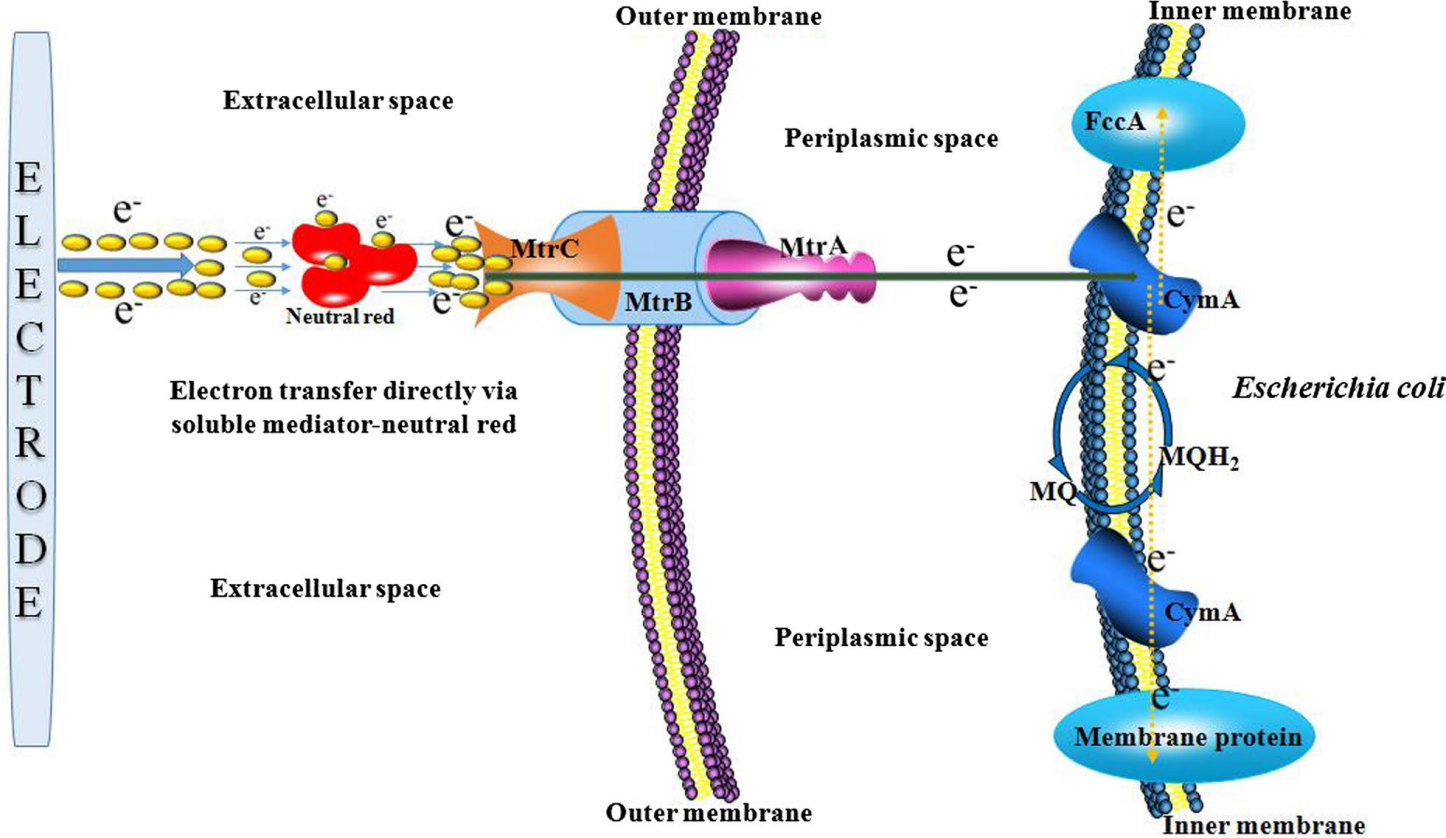
A schematic representation of the electroactive *E. coli* constructed in this research. The heterologously expressed MtrABC complex was assembled in the inner membrane, which allowed the input of electrons transferred from electron carriers. The electrons were delivered to menaquinone or other biological carriers inside the cell, and thus transformed into reducing power for biosynthesis reactions

Previous reports have demonstrated the successful heterologous expression of *mtrC*, *mtrA* and *mtrB* of *S. oneidensis* MR-1 in *E. coli* [25–27]. Moreover, the localization of MtrA, MtrB and MtrC in *E. coli* was similar to that in *S. oneidensis* MR-1 [18, 28–30]. Furthermore, CymA from *S. oneidensis* MR-1 was functionally expressed in *E. coli*, and the membrane-bound protein NapC of *E. coli* was found to probably have a similar function to CymA [31, 32]. These two proteins both transfer electrons from menaquinol to the periplasmic terminal electron acceptor complex [33]. Thus, we designed an electrosynthetically active *E. coli* cell as illustrated in Fig. 3. In the theoretical model, the heterologously expressed MtrABC complex was assembled in the *E. coli* membrane, which allowed the input of electrons transferred from electron carriers. Afterwards, the electrons inside the cell were delivered to menaquinone or other biological carriers inside the cell and transformed into reducing power for biosynthesis reactions.

To determine if the heterologous proteins MtrA, MtrB, MtrC, CymA, and FccA were successfully expressed in *E. coli* cells and presented in the membrane compartment, three experiments were performed including fluorescent tag location tracing, SDS-PAGE of extracted membrane proteins, and protein mass spectrometry of membrane proteins. To analyze cellular localization of the expressed membrane proteins, RFP protein was used as a reporter protein. The genes of membrane proteins were fused with the *rfp* gene respectively for making reporter fusions. The strain *E. coli* T110 without expressing the membrane protein from *S. oneidensis* was used as the control, which only expressed the RFP reporter protein. And the strain *E. coli* T110(pMtrA-RFP), T110(pCymA-RFP) and T110(pFccA-RFP), which had MtrA, CymA and FccA fused with RFP respectively were the experimental group, and the constructed plasmid maps were as shown in Additional file 1: Fig. S5. The fluorescence microscope Leica™ DM5000B was used for measuring RFP fluorescence at respective excitation and emission wavelengths of 557 and 590 nm, using a 100× oil-immersion objective. However, the quality of the fluorescence microscopy images is not ideal. We have repeated the practice for a few times, but the best fluorescent images we could obtain was still somewhat vague. Thus, we did not put these images as formal manuscript figures but left them in supplemental files as shown in Additional file 1: Fig. S6. In Additional file 1: Fig. S6a, the control without membrane protein showed RFP filled with the whole cell, indicating that there is no membrane localization occurred. However, in Additional file 1: Fig. S6b–d, we could see that RFP fluorescence was more distributed towards the edge of cells. The fluorescent microscopy results indicated these membrane proteins was possibly located with the membrane compartment. Since the image quality is low, we had to perform the following experiments to illustrate their location status.

SDS-PAGE of proteins associated with membranes were performed for determining the expression of MtrA, MtrB, MtrC, CymA and FccA, and the result was as illustrated in Additional file 1: Fig. S7. We could see that the control strain *E. coli* T110 almost had no expression of these proteins, whereas, the strain *E. coli* T110(pMtrABC, pFccA-CymA) had relatively clear bands matched the sizes of MtrA, MtrB, MtrC, CymA and FccA.

The protein mass spectrometry was conducted for further determine the expression of the heterologous membrane proteins derived from *S. oneidensis*. The result of membrane protein mass spectrometry was as illustrated in Additional file 1: Fig. S8, the detailed identification results of the MtrA, MtrB, MtrC, CymA and FccA proteins were marked in red in Additional file 1: Fig. S8. It was illustrated that while no MtrA, MtrB, MtrC, CymA and FccA were found in the control samples of *E. coli* T110, all these heterologous proteins scored a “High” in the “Found in Sample” section by the strain *E. coli* T110(pMtrABC, pFccA-CymA), which indicated the presence of these proteins. With the above three experiments, heterologous proteins MtrA, MtrB, MtrC, CymA and FccA were proved to be successfully expressed and presented in the membrane compartment.

**Fig. 4:**
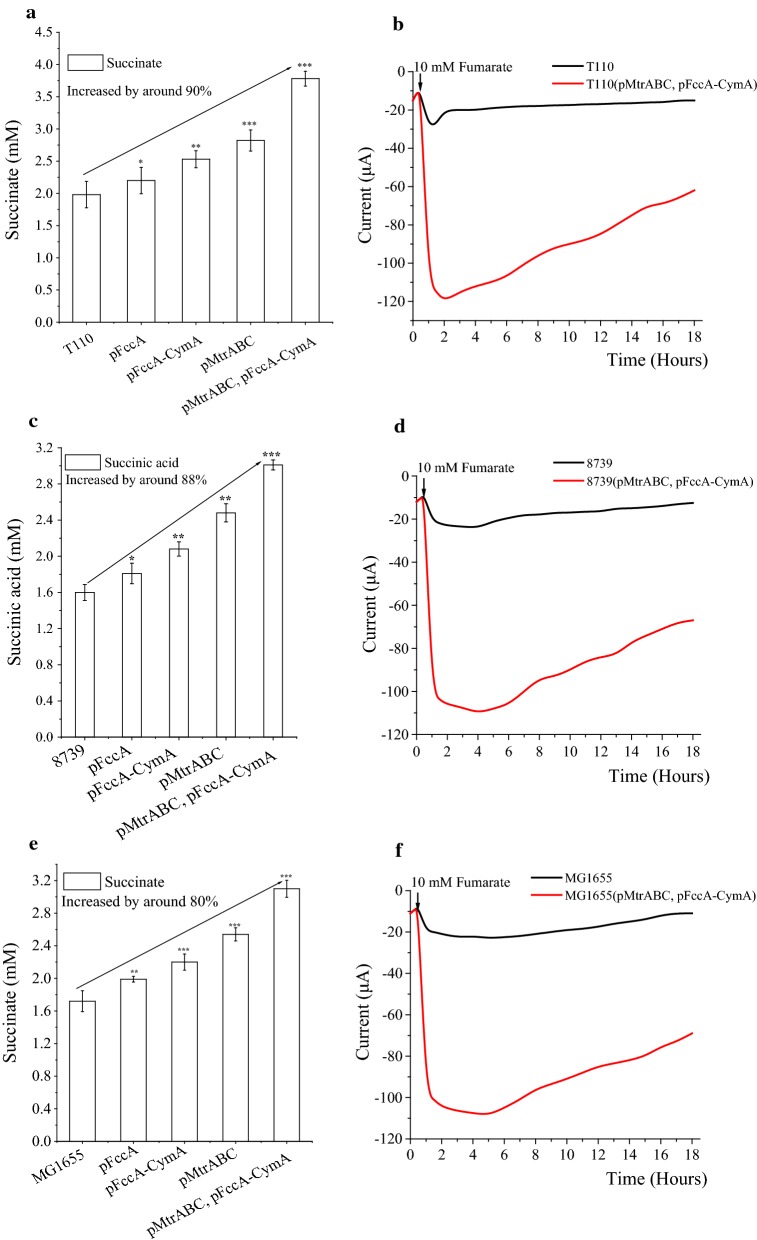
The succinate production of various engineered *E. coli* strains with fumarate as the substrate. **a** Succinate production of *E. coli* T110 expressing different combinations of genes derived from *S. oneidensis* MR-1 in the MES reaction. **b** The i–t curve (current versus time) of the MES system for fumarate reduction catalyzed by *E. coli* T110 and T110(pMtrABC, pFccA-CymA). The Negative electrode was poised at -0.65 V against the Ag/AgCl reference electrode. After 30 min, 10 mM fumarate was added into the MES medium. **c** Succinate production of *E. coli* 8739 expressing different combinations of genes derived from *S. oneidensis* MR-1 in the MES reaction. **d** The i–t curve (current versus time) of the MES system for the reduction of fumarate catalyzed by *E. coli* 8739 and 8739(pMtrABC, pFccA-CymA). **e** Succinate production by *E. coli* MG1655 expressing different combinations of genes derived from *S. oneidensis* MR-1 in the MES reaction. **f** The i–t curve (current versus time) of the MES system for fumarate reduction catalyzed by *E. coli* MG1655 and MG1655(pMtrABC, pFccA-CymA). The data represent the means of three experiments and the error bars represent their standard deviation. The significance of differences was calculated using Student’s *t*-test, asterisks indicate a significant difference compared with the control (***p < 0.001; **p < 0.01; *p < 0.05)

To investigate the effects of the key elements for the cells’ electric activity, different combinations of genes derived from *S. oneidensis* MR-1 were tested in *E. coli* T110 (Fig. 4a). Similarly, the production of succinate via reduction of fumarate was used as a measure of the degree of electric activity of the engineered cells, as well as the system’s overall electric current. As expected, compared to the control strain T110, all engineered strains, including T110(pFccA), T110(pFccA-CymA), T110(pMtrABC) and T110(pMtrABC, pFccA-CymA), exhibited increased succinate production. The highest succinate production was obtained from strain T110(pMtrABC, pFccA-CymA), which displayed a very significant increase of almost 90% (*p *< 0.001) over the parent strain. As shown in Fig. 4b, a significantly increased current was observed with strain *E. coli* T110(pMtrABC, pFccA-CymA) when 10 mM fumarate was added into the MES reaction medium at 0.5 h. The current started decreasing slowly from 2.0 h, and very slowly converged to levels similar to those of the control strain T110, which only exhibited a slowly increasing current from 0.5 h with the addition of fumarate. These results demonstrated that the engineered strain *E. coli* T110 (pMtrABC, pFccA-CymA) expressed all the necessary genes from *S. oneidensis* MR-1, and thus displayed a significantly improved electrical activity. We also tested MES medium with no *E. coli* inoculation as a negative control, and it had no current response with or without fumarate supplementation, as expected.

To further improve the engineering strategy and increase the electric activity of other *E. coli* strains, the representative wild-type strains *E. coli* ATCC8739 and MG1655 were modified in the same way as T110 (pMtrABC, pFccA-CymA), and MES reactions were performed using the resulting engineered strains under the same conditions. As shown in Fig. 4c and d, the engineered strains *E. coli* 8739 (pFccA), 8739 (pFccA-CymA), 8739 (pMtrABC), and 8739 (pMtrABC, pFccA-CymA) showed almost the same performance as their counterparts based on the T110 chassis. The succinate production of ATCC8739 (pMtrABC, pFccA-CymA) increased by nearly 88%, and the difference was very significant (*p *< 0.001). The same was observed for the strains derived from *E. coli* MG1655 (Fig. 4e, f), whereby the succinate production of MG1655(pMtrABC, pFccA-CymA) was increased by 80% compared with the parent strain, and the increase was highly significant (*p *< 0.001). A similar trend of increased succinate production and system-wide current of the three groups of strains indicated that this metabolic engineering strategy is probably universally applicable to *E. coli*.

By calculating the electron balance, as known as the coulombic efficiency, in the MES system (Additional file 1: Table S2), the values of the coulombic efficiency of these processes were at the range of 100–300%, which indicated that the mole value of electrons delivered by the current to the reduced fermentation products did not stoichiometrically account for the increased succinate. In fact, the increased succinate mole value was larger than electrons delivered to the system, similar to the data presented by some other reports [4, 34, 35]. A possible hypothesis was that only a portion of succinate was directly reduced by the accepted electrons, while the other portion of succinate production was due to the cell response to the low redox-potential environment induced by the delivering of the electrons, which led to the increased succinate production from fumarate compared with the condition without electricity in the MES system [4, 34, 35].

### *E. coli* 8739(pMtrABC, pFccA-CymA) was able to perform MES with glucose as the carbon source

Bioelectrochemical techniques offer a promising way to supply reducing power to intracellular electron carriers, such as NADH, by providing electrons through a cathode, enabling the biotransformation and biosynthesis of a number of reduced products [36, 37].

In previous experiments, the electric activity of *E. coli* 8739(pMtrABC, pFccA-CymA) was evaluated using the simple one-step fumarate reduction. To perform a more presentative MES reaction, glucose was used as the carbon source. The supplemented 55.56 mM glucose was totally consumed at room temperature within 7 days with and without electricity, and the resulting fermentation products, including succinate, lactate and ethanol were analyzed (Table 2). With no electricity in the MES system, *E. coli* 8739(pMtrABC, pFccA-CymA) performed a normal mixed-acid fermentation with acetate and ethanol as the major fermentation products at similar molar ratios. When the MES system was supplied with electricity, the fermentation product spectrum was significantly shifted towards the more reduced end. The molar proportion of reduced products, including lactate and ethanol, was significantly improved, while the proportion of the oxidized product acetate decreased at the same time. The lactate yield increased 2.2-fold from 0.10 to 0.22 mol/mol glucose, and ethanol increased 1.5-fold from 0.63 to 0.96 mol/mol glucose.

**Table 2:** MES fermentation profile of *E. coli* ATCC8739(pMtrABC, pFccA-CymA)

Characteristics	Concentration (mM) of the following fermentation products				
	Succinate	Lactate	Formate	Acetate	Ethanol
8739 (pMtrABC, pFccA-CymA) without electricity	1.31 ± 0.02^a^	5.43 ± 0.03	1.06 ± 0.02	32.21 ± 0.50	34.68 ± 0.70
Yield (mol/mol Glucose)	0.02 ± 0.00	0.10 ± 0.00	0.02 ± 0.00	0.58 ± 0.04	0.63 ± 0.05
8739 (pMtrABC, pFccA-CymA) with electricity	2.17 ± 0.01	11.96 ± 0.20	1.60 ± 0.04	24.53 ± 1.00	53.31 ± 1.60
Yield (mol/mol Glucose)	0.04 ± 0.01	0.22 ± 0.01	0.03 ± 0.00	0.44 ± 0.08	0.96 ± 0.08

55.56 mM glucose was consumed in each MES fermentation, room temperature, 200–250 rpm, 7 days, constant sparging with pure N_2_ to ensure strict anaerobic conditions in the MES system

^a^The data is the mean of three replicates, present with standard deviation

A successful MES reaction was thus performed using the electroactive *E. coli* 8739(pMtrABC, pFccA-CymA), and electricity was utilized in the MES system to produce more reduced fermentation products from glucose. The results suggested that the cells in the experimental group supplemented with electricity obtained excess reducing power that was not available to the control group. Therefore, more reduced metabolites were produced to consume the excess reducing power and balance the intracellular redox state [38, 39]. The intracellular reducing power is stored in the form of reduced cofactors such as NADH, FAD and CoQ, indicating that some of the cofactors had accepted electrons and were reduced with supplementation of electricity in the MES system [40–43].

During this electrosynthesis fermentation process, a charge of 69.6 ± 5.5 °C was transferred to the anode, equal to 0.72 ± 0.06 mM of electrons, and the increased reduced products had a total mole amount of 4.55 mM (Table 2). Thus, the coulombic efficiency MES reaction of *E. coli* 8739(pMtrABC, pFccA-CymA) was calculated to be 632%. Similar to the previous fumarate reduction MES reactions, the coulombic efficiency was larger than 100% again. We hypothesized that the electricity-driven reducing power might affect the intracellular redox potential and hence metabolite profiles, and facilitated a higher production of reduced metabolites products, such as succinate, lactate, ethanol [4, 35, 44, 45].

### The electroactive *E. coli* T110 produced succinate at high yield with CO_2_ supplementation in the MES system

**Fig. 5:**
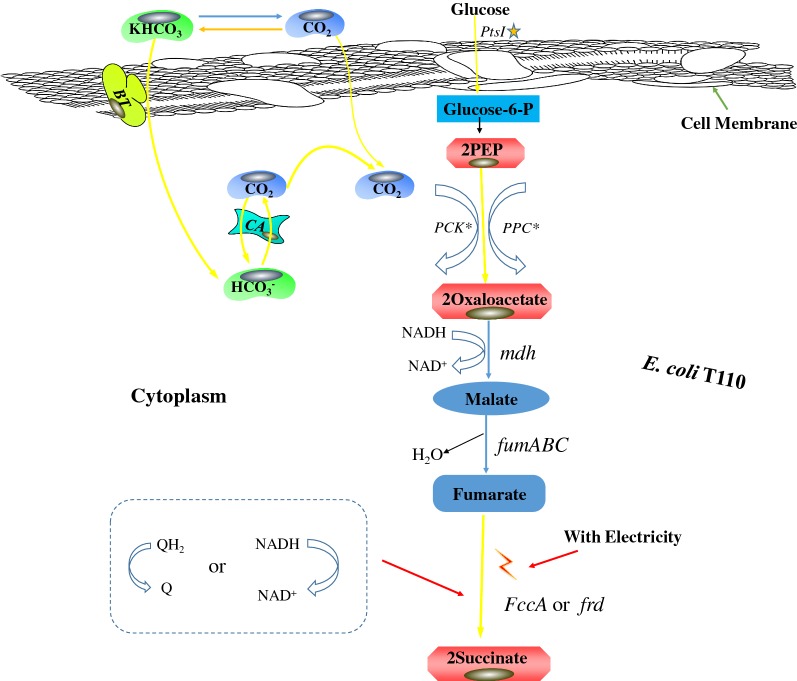
The Succinate synthesis pathway in the electroactive *E. coli* T110 assisted by CCM. A succinate synthesis pathway was constructed mainly by eliminating competing fermentation pathways and activating phosphoenolpyruvate carboxykinase. Using CCM, PCK and PPC were supplemented with increased amounts of CO_2_. In the electroactive *E. coli* T110 chassis, electricity from the MES system was utilized for increased succinate production

In our previous work, the succinate-producing strain *E. coli* T110 was constructed mainly by eliminating competing fermentation pathways and activating phosphoenolpyruvate carboxykinase [46] and the succinate anaerobic fermentation pathway was as exhibited in Additional file 1: Fig S2. In the succinate synthesis pathway, CO_2_ is fixed in a reaction with phosphoenolpyruvate (PEP) catalyzed by PEP carboxykinase, in which 1 mol of glucose can yield 2 mol of succinate with supplementation of CO_2_, considering only the carbon balance. However, due to the limitation of internally generated reducing equivalents, the actual yield is much lower. To achieve a higher succinate yield from glucose, internal reducing power was increased by manipulating the metabolic networks, which proved that reducing power was indeed the major obstacle to achieving the maximal succinate yield [47]. Thus, transforming supplemental substrates or energy into reducing power for succinate production might be a good strategy to increase the succinate production, along with CO_2_ incorporation efficiency. Consequently, an electroactive T110 derivative was engineered to perform the MES with CO_2_ supplementation to achieve production of succinate with high yield in this study (Fig. 5).

The MES reaction catalyzed by the electroactive *E. coli* 8739(pMtrABC, pFccA-CymA) proved the effectiveness of this engineering strategy, and demonstrated the ability of the engineered *E. coli* to perform MES in our system. In this MES reaction, KHCO_3_ was employed to supply the electroactive *E. coli* T110 with CO_2_ and HCO_3_^+^, and electricity was used as a source of supplemental reducing power (Fig. 5). In all test groups, the 27.78 mM glucose supplemented to the media was depleted in the MES system within 7 days at room temperature. In the control groups without electricity, all three strains had similar succinate yields of around 0.6 mol succinate/mol glucose, with the by-product acetate reaching around 0.5 mol/mol glucose (Table 3). Nevertheless, when provided with electricity, the engineered strains achieved significantly higher succinate yields. T110(pMtrABC, pFccA-CymA) produced 26.39 mM succinate with a yield of 0.95 mol/mol glucose, which was respectively 42.3% and 39.7% higher than that of the parent strain *E. coli* T110.

**Table 3:** MES fermentation profile of *E. coli* T110, T110 (pMtrABC, pFccA-CymA) and T110 (pMtrABC, pFccA-CymA, pBTCA)

Characteristics	Consumed Glucose (mM)	Product concentration (mM) and yields without electricity		Product concentration (mM) and yields with MES	
		Succinate	Acetate	Succinate	Acetate
T110	27.28 ± 0.52	16.64 ± 0.36^a^	13.47 ± 0.12	18.55 ± 0.38	13.91 ± 0.07
Yield (mol/mol glucose)		0.61 ± 0.05	0.48 ± 0.03	0.68 ± 0.05	0.51 ± 0.03
T110 (pMtrABC, pFccA-CymA)	27.78 ± 0.52	15.56 ± 0.40	14.68 ± 0.32	26.39 ± 1.20	11.11 ± 0.75
Yield (mol/mol glucose)		0.56 ± 0.03	0.53 ± 0.02	0.95 ± 0.04	0.40 ± 0.02
T110 (pMtrABC, pFccA-CymA, pBTCA)	27.78 ± 0.52	18.06 ± 0.56	14.10 ± 0.50	30.56 ± 1.26	9.16 ± 0.20
Yield (mol/mol glucose)		0.65 ± 0.08	0.51 ± 0.04	1.10 ± 0.03	0.33 ± 0.01

100 mM KHCO_3_ was added into each MES fermentation using 27.78 mM glucose as the substrate, room temperature, 200–250 rpm, 7 days, constant sparging with pure N_2_ to ensure strict anaerobic conditions in the MES system

^a^The data is the mean of three replicates, present with standard deviation

**Fig. 6:**
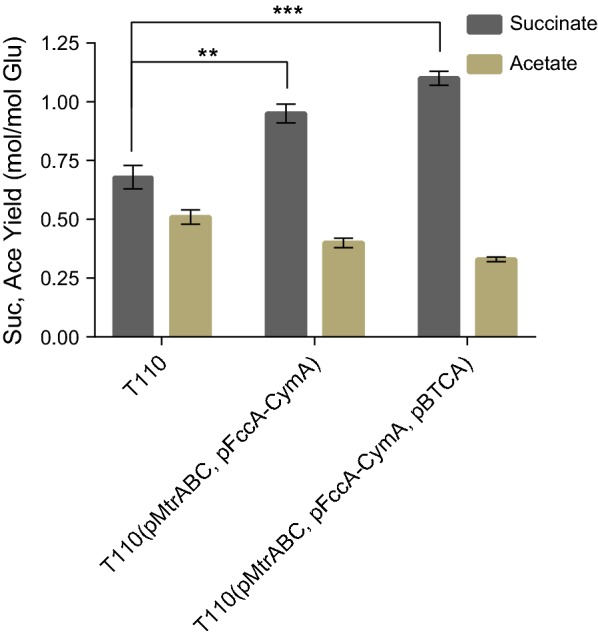
Succinate and acetate yields of *E. coli* T110, T110(pMtrABC, pFccA-CymA) and T110(pMtrABC, pFccA-CymA, pBTCA) fermented in the MES system. The data represent the means of three experiments and the error bars represent their standard deviations. The significance of differences was determined using Student’s *t*-test and presented as *p*-values. The asterisks indicate a significant difference between the mean values of the control and test samples (***p < 0.001; **p < 0.01; *p < 0.05). Suc, succinate; Ace, acetate; Glu, glucose

Considering that the CO_2_ concentration might become a limiting factor in the succinate production process via MES, the intracellular CO_2_ was increased by employing a carbon-concentration mechanism (CCM) derived from cyanobacteria, which was proved to be functional in *E. coli* [48]. To do so, the bicarbonate transporter (*BT*) and carbonic anhydrase (*CA*) genes from *Synechococcus* sp. PCC7002 were overexpressed simultaneously in T110(pMtrABC, pFccA-CymA) [49, 50]. As illustrated in Fig. 6 and Table 3, the resulting strain T110(pMtrABC, pFccA-CymA, pBTCA) showed an even more prominent improvement of succinate production. It produced 30.56 mM succinate with a yield 1.10 mol/mol glucose, which represented 64.7% and 61.8% increases over the original strain *E. coli* T110, respectively. Statistical analysis of the results revealed a significantly higher succinate yield in this strain than in the parent T110 (*p *< 0.001). During this electro-fermentation process, a charge of 72.6 ± 6.5 °C was transferred to the anode in the MES system, which corresponding to 0.75 ± 0.07 mM of electrons. The coulombic efficiency of increased succinate by *E. coli* T110 was determined to be 50.7%, and that of *E. coli* T110(pMtrABC, pFccA-CymA) and T110(pMtrABC, pFccA-CymA, pBTCA) was 287.8% and 332.2% respectively. The results thus demonstrate that the introduction of a CO_2_ delivery system is an effective strategy to increase the succinate yield of electroactive *E. coli* cell factories.

## Conclusions

In this research, an electroactive succinate-producing cell factory, *E. coli* T110(pMtrABC, pFccA-CymA, pBTCA), was obtained by both internal metabolic-network engineering and the introduction of electroactivity, mainly in the periplasm compartment (Figs. 3 and 5). To achieve the maximal succinate yield from glucose, excess reducing power was needed in the succinate fermentation. Thus, we designed an electrosynthetically active *E. coli* cell as illustrated in Fig. 3, in which the heterologously expressed MtrABC complex was assembled, which allowed the input of electrons transferred from electron carriers. Afterwards, the electrons were delivered to menaquinone or other biological carriers inside the cell, and transformed into usable reductive power for biosynthesis reactions.

The best strain T110(pMtrABC, pFccA-CymA, pBTCA) was fermented in an MES system supplemented with HCO_3_^+^, with neutral red as the electron carrier, and achieved a succinate yield of 1.10 mol/mol glucose—a 1.6-fold improvement over the parent strain T110. This strain is to our best knowledge the first electroactive microbial cell factory engineered to directly utilize electricity for the production of a specific product. Due to the versatility of the *E. coli* platform, this research can most certainly be exploited for the engineering of various other cell factories that utilize electricity for bioproduction. Since CO_2_ fixation is a biosynthesis reaction that requires vast energy inputs, the application of our MES system may provide a solution for the conversion of CO_2_ into various bio-based chemicals and fuels. A further implementation of this research might enable the commercialization of MES technologies [9, 51].

## Additional file

**Additional file 1: Table S1.** Primers used in this study. **Table S2.** The succinate production by electroactive *E. coli* T110, T110(pMtrABC, pFccA-CymA), 8739, 8739(pMtrABC, pFccA-CymA), MG1655, MG1655(pMtrABC, pFccA-CymA) without electricity in the MES system using 10 mM fumarate as the sole carbon source. **Figure S1.** The microbial electrosynthesis (MES) system built and used in this research. **Figure S2.** Overview of the mixed acid fermentation pathway by *E. coli* T110 under anaerobic condition. **Figure S3.** Determination of optimal concentration of neutral red addition by *E. coli* 8739. **Figure S4.** The effect of different neutral red concentrations on the current levels with *E. coli* T110(pMtrABC, pFccA-CymA). **Figure S5.** The plasmid maps of RFP fusion proteins. **Figure S6.** The fluorescent images of RFP fluorescence of control strain *E. coli* T110, and *E. coli* T110(pMtrA-RFP), T110(pCymA-RFP) and T110(pFccA-RFP) that expressed membrane proteins of MtrA, CymA and FccA fused with RFP reporter protein. **Figure S7.** SDS-PAGE of membrane proteins extracted from the control strains *E. coli* T110 and *E. coli* T110(pMtrABC, FccA-CymA). **Figure S8.** The protein mass spectrometry for the determination of membrane proteins by the strains *E. coli* T110 and *E. coli* T110(pMtrABC, pFccA-CymA).
