# Supplementary material for: Engineering an electroactive Escherichia coli for the microbial electrosynthesis of succinate from glucose and CO2
Source: Microb Cell Fact. 2019 Jan 28;18:15. doi: 10.1186/s12934-019-1067-3 (PMC6348651; doi:10.1186/s12934-019-1067-3)

**Table S1** Primers used in this study

| **Prime name** | **Sequence** |
| --- | --- |
| **Construction of pMtrABC** | |
| pACYC184-K-F | CCAGGTCTCAGTAATGCGTTTCTTTAATTAAGGGAGAGCC |
| pACYC184-K-R | CCAGGTCTCAATGGTATATCTCCTTGAATTCCATGGTCTGTTTCC |
| MtrA-F | CCAGGTCTCACCATGAAGAACTGCCTAAAAATGAAAAACCTACTGC |
| MtrA-R | CCAGGTCTCATATTAGCGCTGTAATAGCTTGCCAGATGGATGG |
| MtrB-F | CCAGGTCTCAAATAAGAAGGAGATATACCGAGAAAATGAAATTTAAA  CTCAATTTGATCACTC |
| MtrB-R | CCAGGTCTCATAGCAAATGGATTAGAGTTTGTAACTCATGCTCAG |
| MtrC1-F | CCAGGTCTCAGCTAAGAAGGAGATATACCATGATGAACGCACAAA  AATCAAAAATCGC |
| MtrC1-R | CCAGGTCTCAGGCATGTCGGCTTCGTTAGTGGC |
| MtrC2-F | CCAGGTCTCATGCCAGTGATTGGCCTCGCAAATTTAG |
| MtrC2-R | CCAGGTCTCATTACATTTTCACTTTAGTGTGATCTGC |
| **Construction of pFccA-CymA** | |
| pTrac99A-K-F | CCAGGTCTCAACTGTCAGACCAAGTTTACTCATATATAC |
| pTrac99A-K-R | CCAGGTCTCATATGGTATATCTCCTTGAATTCCATGGTC |
| FccA-F | CCAGGTCTCACATATGTTCACAAGAAAGATTCAAAAAACAGCACTAGC |
| FccA-R | CCAGGTCTCAATTTAATTATCTTTAGCGAATTTAGCGGCAGATGCACC |
| CymA-F | CCAGGTCTCAAAATTAAAGAGGAGAATACTAGATGAACTGGCGTGC  ACTATTTAAACCCAGC |
| CymA-R | CCAGGTCTCACAGTTTTATCCTTTTGGATAGGGGTGAGCGACACC |
| **Construction of pFccA** | |
| pTrac99A-spe-F | CCAGGTCTCAAACTGTCAGACCAAGTTTACTCATATATACTTTAG |
| pTrac99A-spe-R | CCAGGTCTCAGCGGCTTGAACGAATTGTTAGACATTATTTGCC |
| pTrac99A-M-F | CCAGGTCTCACCGCCTTTTTACGGTTCCTGGCCT |
| pTrac99A-M-R | CCAGGTCTCAATAATTCTGTTTTATCAGACCGCTTCT |
| pTrac99A-FccA-F | CCAGGTCTCATTATGTTCACAAGAAAGATTCAAAAAACAGCACTAGC |
| pTrac99A-FccA-R | CCAGGTCTCAAGTTAATTATCTTTAGCGAATTTAGCGGCAGATGCACC |
| **Construction of pMtrA-RFP** | |
| 184-F | CCAGGTCTCATGCGTTTCTTTAATTAAGGGAGAGCCTG |
| 184-R | CCAGGTCTCAGGTATATCTCCTTGAATTCCATGGTCTG |
| MtrA1-F | CCAGGTCTCATACCATGAAGAACTGCCTAAAAATGAAA |
| MtrA1-R | CCAGGTCTCACCATGCGCTGTAATAGCTTGCCAGA |
| RFP-F | CCAGGTCTCAATGGCGAGTAGCGAAGACGTTATCAAAG |
| RFP-R | CCAGGTCTCACGCATTAAGCACCGGTGGAGTGACGACC |
| **Construction of pCymA-RFP and pFccA-RFP** | |
| 99A-F | CCAGGTCTCACTGTCAGACCAAGTTTACTCATATATAC |
| 99A-R | CCAGGTCTCAGGTATATCTCCTTGAATTCCATGGTCTG |
| CymA1-F | CCAGGTCTCATACCATGAACTGGCGTGCACTATTTAAA |
| CymA1-R | CCAGGTCTCACCATTCCTTTTGGATAGGGGTGAGCGAC |
| FccA1-F | CCAGGTCTCATACCATGTTCACAAGAAAGATTCAAAAA |
| FccA1-R | CCAGGTCTCACCATATTATCTTTAGCGAATTTAGCGGC |
| RFP-F | CCAGGTCTCAATGGCGAGTAGCGAAGACGTTATCAAAG |
| RFP1-R | CCAGGTCTCAACAGTTAAGCACCGGTGGAGTGACGACC |

**Table S2** The succinate production by electroactive *E. coli* T110, T110(pMtrABC, pFccA-CymA), 8739, 8739(pMtrABC, pFccA-CymA), MG1655, MG1655(pMtrABC, pFccA-CymA) without electricity in the MES system using 10 mM fumarate as the sole carbon source

| Strains | Succinate production (mM) in the BES system | |
| --- | --- | --- |
|  | Without electricity | With electricity |
| T110 | 1.83 ± 0.03 | 1.89 ± 0.04 |
| T110(pMtrABC, pFccA-CymA) | 1.90 ± 0.04 | 3.78 ± 0.10 |
| 8739 | 1.51 ± 0.02 | 1.60 ± 0.02 |
| 8739(pMtrABC, pFccA-CymA) | 1.49 ± 0.01 | 3.01 ± 0.05 |
| MG1655 | 1.55 ± 0.01 | 1.72 ± 0.01 |
| MG1655(pMtrABC, pFccA-CymA) | 1.52 ± 0.02 | 3.10 ± 0.06 |

Each value is the mean of three parallel replicates ± standard deviation

**Figure S1** The microbial electrosynthesis (MES) system built and used in this research


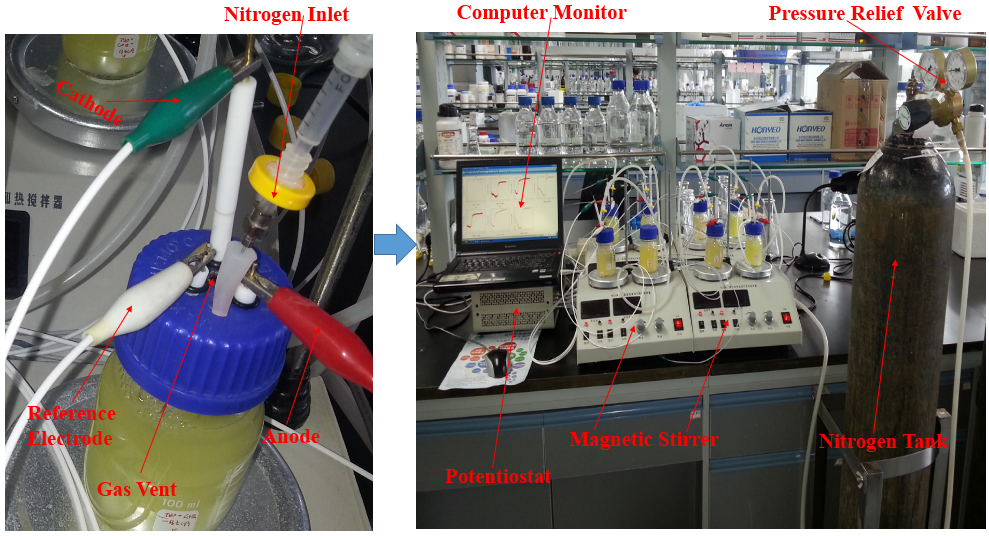


**Figure S2** Overview of the mixed acid fermentation pathway by *E. coli* T110 under anaerobic condition

 Formate, acetate, ethanol, lactate, succinic acid biosynthesis pathways were included under anaerobic conditions in *E. coli* T110. PEP, phosphoenolpyruvate. Yellow stars indicated metabolic reactions that had been blocked through gene deletion methods. The pathway of *E. coli* T110 including these yellow stars.

**Figure S3.** Determination of optimal concentration of neutral red addition by *E. coli* 8739
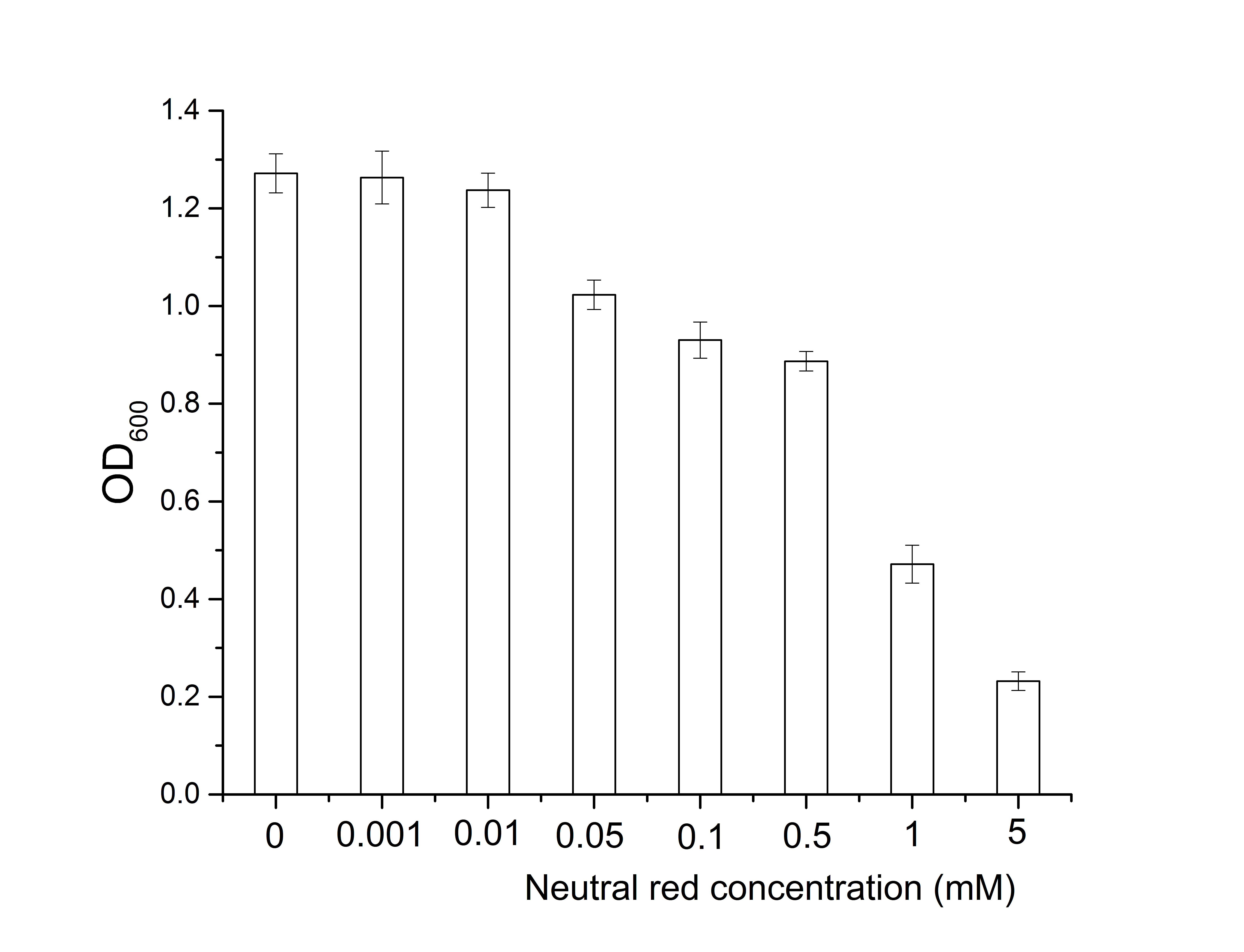


The optimal concentration determination of the electron carrier of neutral red by *E. coli* 8739. The data represent the means of three parallel replicates and the error bars represent the standard deviation.

**Figure S4.** The effect of different neutral red concentrations on the current levels with *E. coli* T110(pMtrABC, pFccA-CymA)


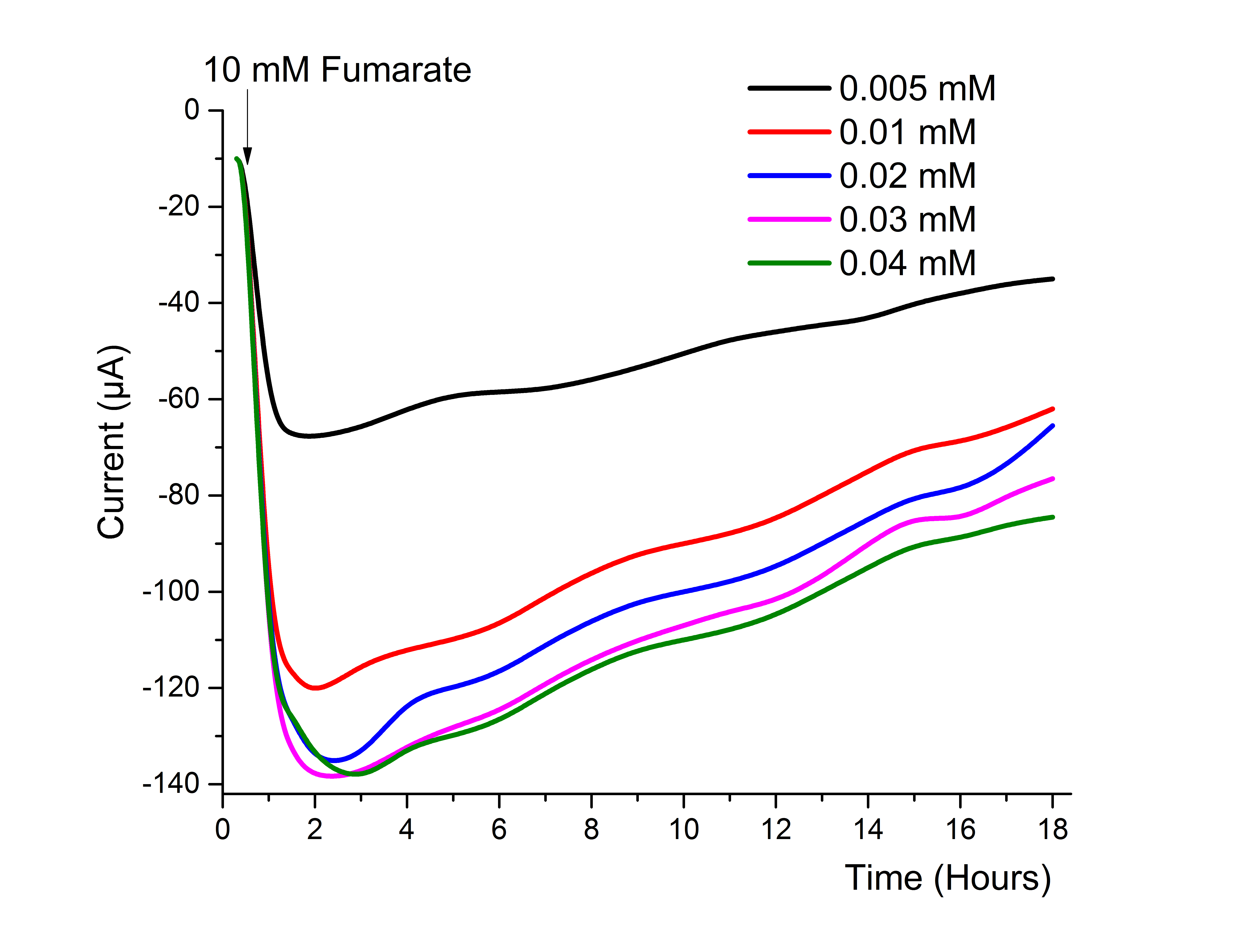


**Figure S5.** The plasmid maps of RFP fusion proteins


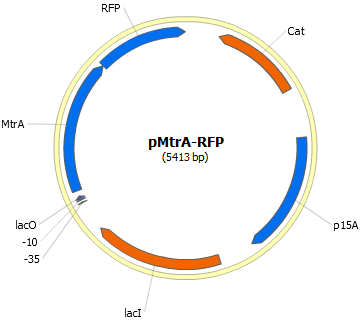


The fusion protein of MtrA with reporter protein RFP


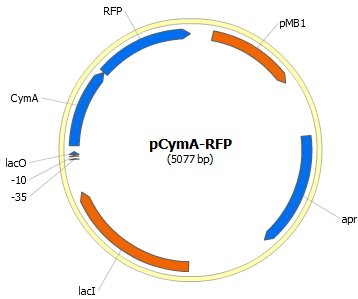


The fusion protein of CymA with reporter protein RFP


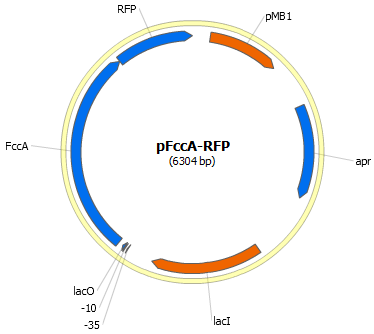


The fusion protein of FccA with the reporter protein RFP

**Figure S6.** The fluorescent images of RFP fluorescence of control strain *E. coli* T110, and *E. coli* T110(pMtrA-RFP), T110(pCymA-RFP) and T110(pFccA-RFP) that expressed membrane proteins of MtrA, CymA and FccA fused with RFP reporter protein


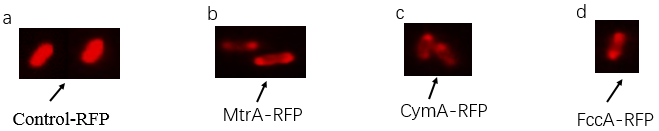


**Figure S7.** SDS-PAGE of membrane proteins extracted from the control strains *E. coli* T110 and *E. coli* T110(pMtrABC, FccA-CymA)


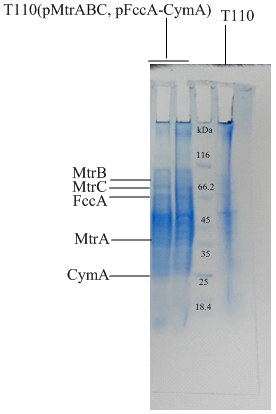


**Figure S8.** The protein mass spectrometry for the determination of membrane proteins by the strains *E. coli* T110 and *E. coli* T110(pMtrABC, pFccA-CymA).

The protein mass spectrometry result of the strain *E. coli* T110(pMtrABC, pFccA-CymA)
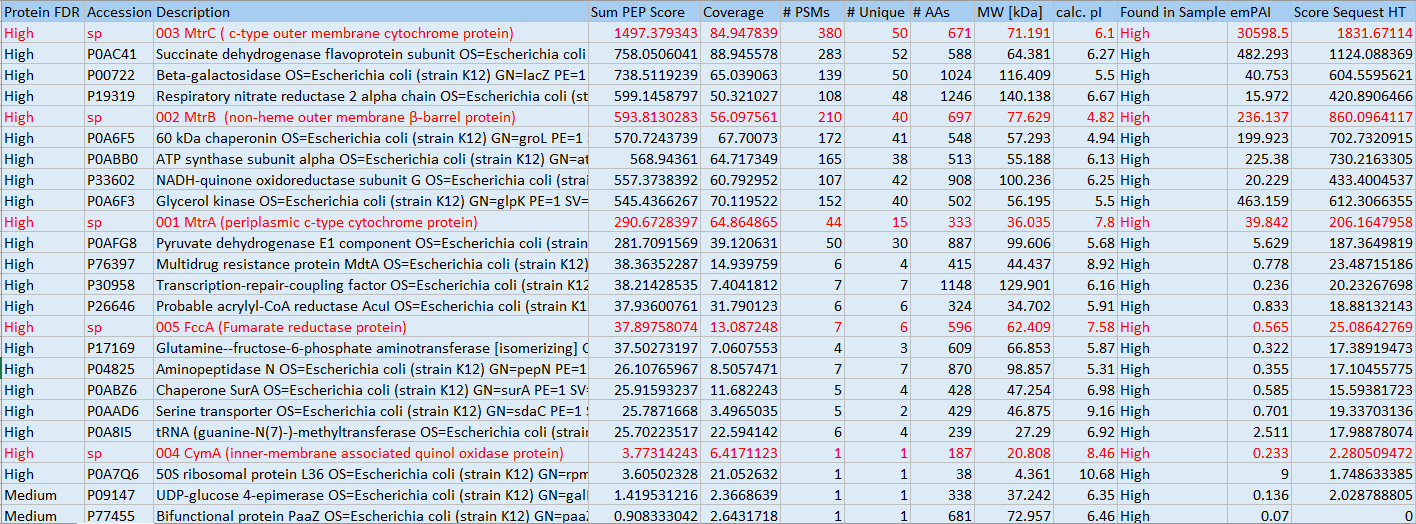


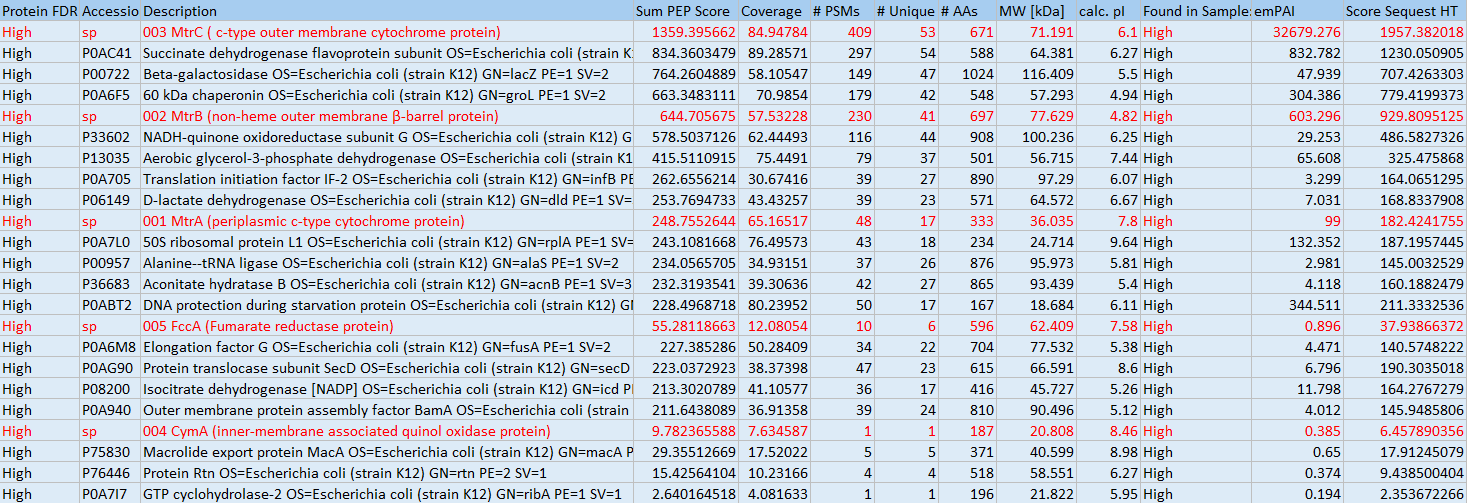


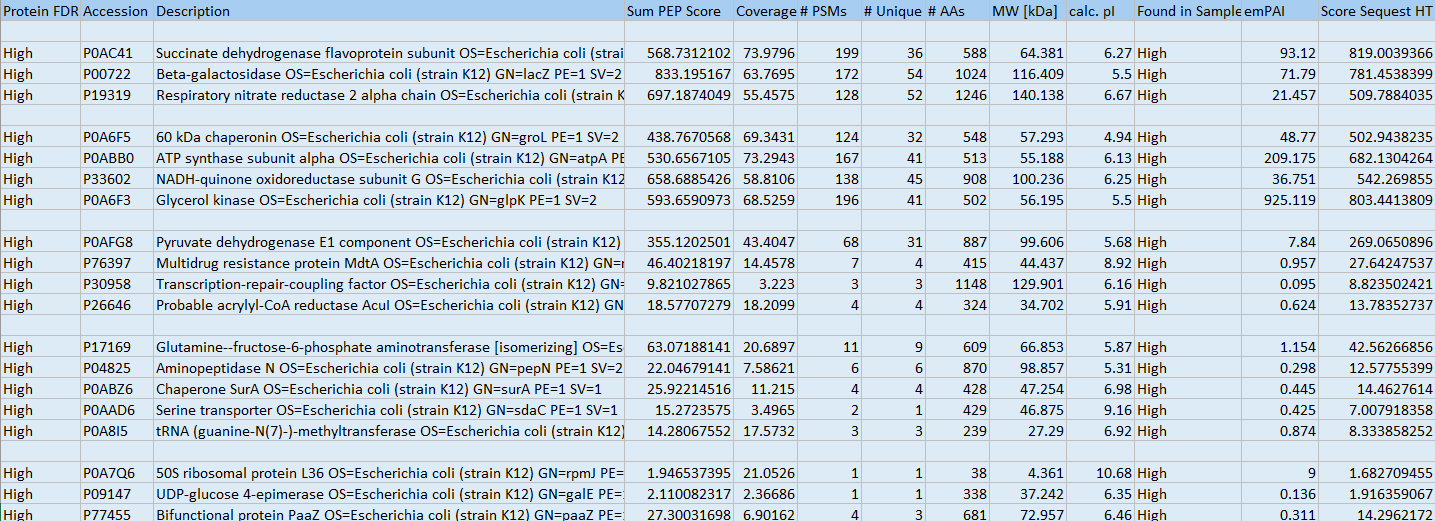
The protein mass spectrometry result of the control strain *E. coli* T110


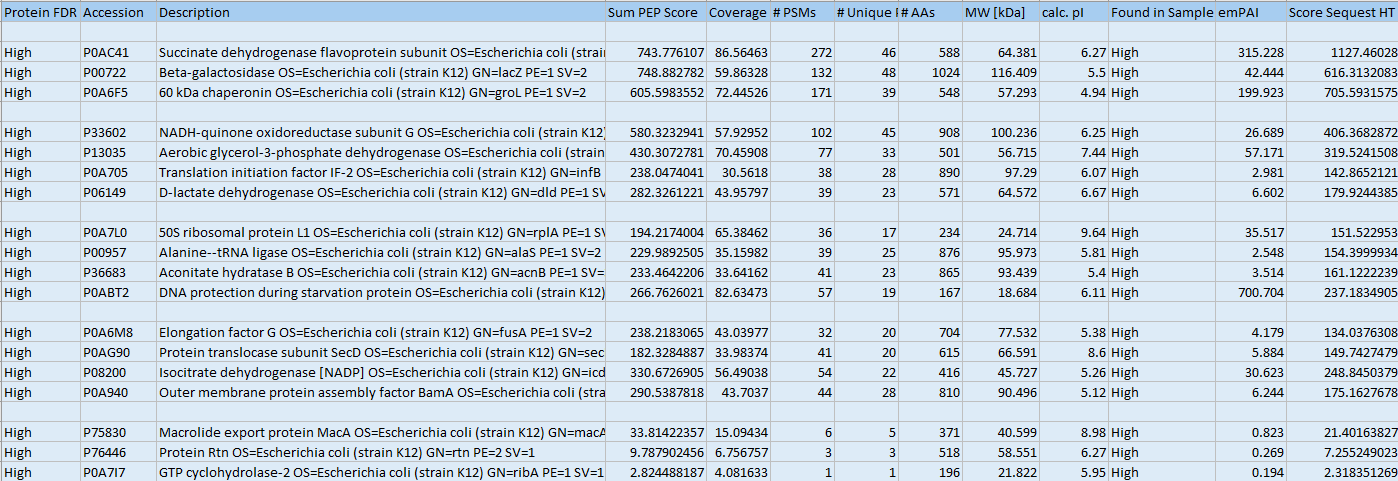

Supplement: Supplementary file 1 — Additional file 1: Table S1. Primers used in this study. Table S2. The succinate production by electroactive E. coli T110, T110(pMtrABC, pFccA-CymA), 8739, 8739(pMtrABC, pFccA-CymA), MG1655, MG1655(pMtrABC, pFccA-CymA) without electricity in the MES system using 10 mM fumarate as the sole carbon source. Figure S1. The microbial electrosynthesis (MES) system built and used in this research. Figure S2. Overview of the mixed acid fermentation pathway by E. coli T110 under anaerobic condition. Figure S3. Determination of optimal concentration of neutral red addition by E. coli 8739. Figure S4. The effect of different neutral red concentrations on the current levels with E. coli T110(pMtrABC, pFccA-CymA). Figure S5. The plasmid maps of RFP fusion proteins. Figure S6. The fluorescent images of RFP fluorescence of control strain E. coli T110, and E. coli T110(pMtrA-RFP), T110(pCymA-RFP) and T110(pFccA-RFP) that expressed membrane proteins of MtrA, CymA and FccA fused with RFP reporter protein. Figure S7. SDS-PAGE of membrane proteins extracted from the control strains E. coli T110 and E. coli T110(pMtrABC, FccA-CymA). Figure S8. The protein mass spectrometry for the determination of membrane proteins by the strains E. coli T110 and E. coli T110(pMtrABC, pFccA-CymA). [file 12934_2019_1067_MOESM1_ESM.docx]
